# Mealtime Anxiety and Coping Behaviour in Parents and Children during Weaning in PKU: A Case-Control Study

**DOI:** 10.3390/nu11122857

**Published:** 2019-11-21

**Authors:** Sharon Evans, Anne Daly, Jo Wildgoose, Barbara Cochrane, Catherine Ashmore, Shauna Kearney, Anita MacDonald

**Affiliations:** 1 Dietetic Department, Birmingham Women’s and Children’s Hospital NHS Foundation Trust, Birmingham B4 6NH, UK; a.daly3@nhs.net (A.D.); catherine.ashmore@nhs.net (C.A.); anita.macdonald@nhs.net (A.M.); 2 Dietetic Department, Bradford Teaching Hospitals NHS Trust, Bradford BD9 6RJ, UK; jo.wildgoose@bthft.nhs.uk; 3 Dietetic Department, Royal Hospital for Children Glasgow, Glasgow G51 4TF, UK; Barbara.cochrane@ggc.scot.nhs.uk; 4 Psychology Department, Birmingham Women’s and Children’s Hospital NHS Foundation Trust, Birmingham B4 6NH, UK; shauna.kearney@nhs.net

**Keywords:** Phenylketonuria (PKU), weaning, mothers, anxiety, stress, coping, Child Health Inventory for Parents (CHIP), Beck Anxiety Inventory (BAI)

## Abstract

Solid food introduction may create anxiety for parents of children with phenylketonuria (PKU) due to the burden associated with protein substitute (PS) administration and natural protein restriction. In a longitudinal, prospective study, 20 mothers of children with PKU and 20 non-PKU control mothers completed 4 questionnaires (mealtime emotions, feed-time, Beck’s anxiety inventory and the coping health inventory for parents), examining parent/child mealtime emotions, anxiety, stress and coping strategies at child ages: weaning start, 8 months (m), 12 m, 15 m, 18 m and 24 m. Overall, mothers of children with PKU cope well with solid food introduction when applying a low-phenylalanine diet, with comparable low levels of stress and anxiety reported in both PKU and non-PKU groups. However, mothers of children with PKU reported peak scores in anxiety for emotive/cognitive symptoms at a child age of 15 m, and higher use of coping strategies at 15 m and 24 m (*p* < 0.05) of age. Generally, there was a trend that maternal anxiety regarding child rejection of PS increased with time, peaking between 12–24 m. In PKU, a child age of 12–18 m is identified as a key period when mothers feel most anxious/stressed with feeding, coinciding with raised blood phenylalanine levels probably associated with teething, illness and developing independence. Health professionals should be conscious of this vulnerable period and be prepared to offer more directional support as required.

## 1. Introduction

A diagnosis of phenylketonuria (PKU), an inherited metabolic amino acid disorder, is a life-changing event for parents [1]. Treatment involves strict adherence to a controlled, very low-protein diet, consisting of a limited range of special low-protein foods in order to maintain blood phenylalanine (Phe) within the target range and prevent neurological sequelae. Parents must ensure that their children consume a non-negotiable and weighed amount of natural protein together with a bitter tasting protein substitute in prescribed amounts each day. Parents understand that the neurological outcome of their child depends on their ability to successfully implement diet therapy, and this can have a significant impact on stress and quality of life [1,2,3,4].

The process of weaning a child with PKU is likely to enhance parental anxiety [3], and whilst anxiety is a normal emotion that prepares the mind and body to respond to a threat, when it persists over long periods, it can lead to a number of negative medical and psychological outcomes including the development of anxiety disorders [5]. The guilt, grief and disappointment of not having a healthy baby is prominent, and they must face administering a severely restrictive diet in order to control their child’s blood Phe levels at a time when they are still emotionally vulnerable [3,5]. There are many milestones that must be reached (e.g., introduction of a measured quantity of Phe from solid food each day, tolerance of a second-stage spoonable protein substitute and acceptance of special low-protein foods), which require extensive parental organisational skills and tenacity. Any food refusal, rejection of protein substitute, child illness, inability to access special dietary products, social situations involving food (parties, eating out) and sub-optimal blood Phe control is likely to heighten parental anxiety [1,3]. Consequently, there may be conflict between maintaining blood Phe levels within the target range and allowing dietary deviations aimed at meeting child and parent psychosocial needs and the desire for family normality [5].

Thereby, parents of children with PKU require a diverse range of complex skills and additional time in order to manage and control dietary treatment, as well as the stress associated with adherence, social isolation and regular blood Phe monitoring [1,3,6,7]. Metabolic and psychological outcomes are likely to be influenced by parental coping strategies and the ability of the family to alter their lifestyle to accommodate the demands of a complex dietary regimen [2,3,4,5]. However, it is uncertain what coping mechanisms exist in the parents of children with PKU, and how this might compare with parents of children with other health conditions or those of normal healthy children.

The aim of this study was to compare maternal and child anxiety and coping mechanisms in PKU with that of mothers of non-PKU children during the first two years of life whilst transitioning onto a solid food diet. This is to determine the effect of the additional burdens of protein substitute administration and natural protein prescription adherence.

## 2. Materials and Methods 

### 2.1. Subjects 

#### 2.1.1. Phenylketonuria (PKU) Group

Twenty children (14 male) diagnosed with PKU at newborn screening and commencing weaning onto solid foods during the 2 year study period were recruited prospectively from three UK specialist PKU centres (Birmingham Children’s Hospital (*n* = 17), Bradford Teaching Hospitals NHS Trust (*n* = 2) and the Royal Hospital for Children, Glasgow (*n* = 1)). Subjects were recruited and reviewed by the same dietitian at each centre. Data was recorded from 4 months for 16 subjects, from 5 months for 19 subjects and from 6 months for all 20 subjects.

#### 2.1.2. Control Group

Twenty control children who were weaning were recruited prospectively (12 male, 18 matched with the PKU group for mother’s educational level and birth order). They included siblings of inherited metabolic disorder (IMD) patients, or children recruited from a local community centre. The caregivers of control subjects received no dietary advice from one of two research dietitians. Data was recorded from 4 months for 9 subjects, from 5 months for 19 subjects and from 6 months for all 20 subjects.

#### 2.1.3. Demographics

All subjects were white Caucasian except for 2 control children of mixed 50% Caucasian, 50% Afro-Caribbean decent. Five children from the PKU group and one from the control group had a sibling with PKU and three carers in both groups were single mothers (note: the control child with a PKU sibling had much older adult siblings so the time lapse since they experienced weaning a child with PKU was several years).

Median age of PKU diagnosis was 10 days (range: 2–16 days). Median age of weaning was 4.3 m (range: 2.9–6.6 m) for PKU and 5.1 m (range: 3.7–6.5 m) for controls (*p* = 0.04). The decision to start introduction of solid foods was initiated by parents/caregivers in both groups. For children with PKU, the prescribed target total protein intake was 3 g/kg/day [8] and the natural protein intake at weaning was a median of 5 g/day (range: 3–7 g/day). 

PKU group respondents utilised support from variable and multiple sources: 100% (*n* = 20) reported the help of a dietitian, 30% (*n* = 6) a health visitor, 25% (*n* = 5) a General Practitioner (GP) or paediatrician, 20% (*n* = 4) a specialist nurse, 50% (*n* = 10) relied on support from their own parents (children’s grandparents), 35% (*n* = 7) from other family members, 50% (*n* = 10) accessed the patient support group (National Society for PKU (NSPKU)) and 65% (*n* = 13) sited the internet (websites/social media).

### 2.2. Study Procedures

This was a longitudinal, prospective, observational case-control study investigating anxiety and stress during mealtimes from age of weaning to 2 years.

Following introduction and establishment of low Phe (protein) solids at 4–6 months of age, children with PKU were gradually weaned from a phenylalanine (Phe)-free liquid infant protein substitute onto a semi-solid spoonable Phe-free weaning protein substitute (PKU Anamix First Spoon; Nutricia), providing 2 g of protein equivalent and 16 kcal per 5 g serving. In parallel, and under the supervision of a clinical dietitian, Phe allocation from breastmilk or infant formula was replaced with equivalent Phe, from weaning foods in the form of a 50 mg exchange system. Parents of both PKU and control children determined the age for weaning based on their assessment of readiness. However, infants with PKU were regularly monitored so there were opportunities for dietitians to discuss and advise about weaning. 

Four questionnaires were completed by mothers of children with PKU and control mothers: (1) mealtime emotions, (2) feed-time, (3) Beck’s anxiety inventory and (4) the child health inventory for parents (CHIP). Mealtime emotions was completed monthly from weaning until 12 months of age, then at 15, 18 and 24 months. This non-validated questionnaire comprised 5 statements with a 5-point Likert scale response, addressing maternal experiences during their child’s mealtimes with respect to how stressed (1)/relaxed (5), and rushed (1)/unrushed (5) they felt, how noisy (1)/quiet(5) mealtimes were and if mealtimes were mostly tearful (1) or happy (5) for both themselves and their child.

The remaining three questionnaires were completed by mothers at weaning commencement, 8, 12, 15, 18 and 24 months of age. The non-validated feed-time questionnaire comprised 9 questions (8 for controls) with a 7-point Likert scale response. Questions addressed maternal feelings before giving their child a bottle feed and before giving solids. For mothers of PKU children, additional questions addressed feelings around administering a weaning Phe-free spoonable protein substitute, including how they felt: immediately before administration, if their child refused, when their child had finished and compared to how they felt three months ago. They were also asked if they ever delayed protein substitute administration because of the way it made them feel and how much they would prefer someone else to administer it to their child. Control mothers were asked the same questions but with reference to how they felt when giving solid foods.

The Beck anxiety inventory (BAI) [9,10] is a validated, 21-item, multiple-choice self-report questionnaire that measures the severity of anxiety, with each question describing emotional, physiological or cognitive symptoms of anxiety, and mothers reported the extent to which they had been concerned by each of the symptoms in the previous week (scoring: not at all, mildly, moderately or severely). Two thirds of the questionnaire (*n* = 14/21) focuses on physiological symptoms of anxiety such as trembling, numbness, dizziness and sweating. A total score of 0–7 indicates minimal anxiety, 8–15 mild anxiety, 16–25 moderate anxiety and 26–63 severe anxiety.

The coping health inventory for parents (CHIP) is a validated questionnaire designed for use with families of children with a serious and/or chronic illness [11]. CHIP lists 45 coping behaviours, each scored from 0 (not helpful) to 3 (extremely helpful). Coping behaviours are divided into 3 subsets that identify different coping patterns: (a) maintaining family integration, cooperation and an optimistic definition of the situation (19 items), (b) maintaining social support, self-esteem and psychological stability (18 items) and (c) understanding the medical situation through communication with other parents and consultation with the medical staff (8 items). The sum/total of each item within subsets provides a measure of the usefulness of that coping strategy in the management of the condition. A high score represents a higher degree of coping. The same questionnaire was used for both mothers of PKU and control children, but during analysis, questions specifically relating to medical care (*n* = 6 from subset 1) were removed from both groups to enable comparison. Data was also compared with normative data provided by the questionnaire authors based on the results of 308 mothers with a chronically ill child [11].

Weekly blood spots for Phe were taken by parents on standard filter cards, Perkin Elmer 226 (UK Standard Newborn Screening (NBS), Public Health England, London, UK). Parents had received training on blood spot collection prior to study commencement. Blood spots were returned to the subject’s hospital laboratories. Blood Phe was analysed by MS/MS tandem mass spectrometry.

Data on growth, qualitative aspects of feeding development, gastro-intestinal symptoms, the feeding environment, feeding practices and progression, and weaning problems, are reported in separate publications [12,13].

### 2.3. Ethical Approval 

This study was conducted according to the guidelines laid down in the Declaration of Helsinki and a favourable ethical opinion was obtained from West Midlands–South Birmingham National Research Ethics Service (NRES) Committee. Written informed consent was obtained from the mothers of all participants.

### 2.4. Data Analysis

As an observational study, there was no formal hypothesis comparing the two treatment groups and therefore, no formal power calculation to determine the study size. The number of subjects recruited was determined by the number of subjects diagnosed with PKU per year in the study centres (approximately 4–6 per centre). Quantitative outcome measures are summarised, and descriptive statistics of the data are presented. Between-group differences for questionnaire responses were analysed using suitable non-parametric tests (paired/unpaired *t*-tests and Mann–Whitney tests) using GraphPad Prism version 6.01 for Windows, GraphPad Software, La Jolla, CA, USA. Correlations between blood Phe results and questionnaire responses were calculated using Spearman’s rank correlation. In addition, data was analysed using longitudinal regression techniques using a linear mixed-models approach, including subject ID as a random intercept, time as a categorical factor and, where appropriate, treatment group (PKU versus control). These were analysed using R version 3 computer software.

## 3. Results

### 3.1. Mealtime Emotions Questionnaire

**Figure 1:**
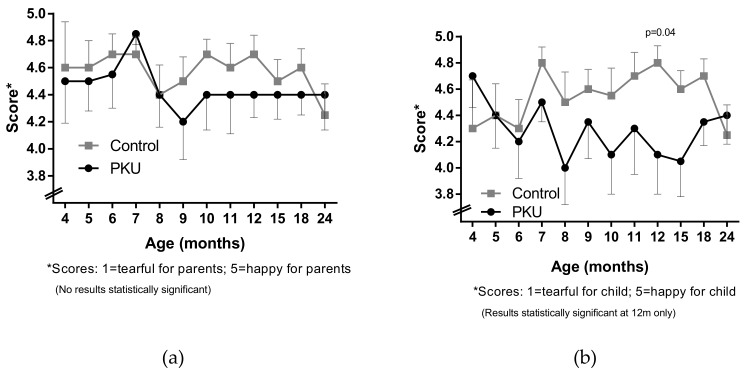
Mean (**a**) maternal and (**b**) child mood during children’s mealtime.

For both PKU and non-PKU groups, levels of stress and mood reported at mealtimes were generally not statistically different over time and were mainly positive (score between 4 to 5 out of 5). However, there was a trend for mealtime mood to be less enjoyable for both parent and child with PKU (Figure 1) between the age of 8 m and 24 m, compared to before 8 m and compared to controls. Children with PKU were reported by mothers to be “less happy” than their control peers, although this only reached significance at 12 m (*p* = 0.04; Mann–Whitney test) (Figure 1b). Longitudinal regression analysis demonstrated that there was a significant difference (*p* = 0.002) between children with PKU and control children in terms of how happy they were at mealtimes, and this difference remained unchanged overtime between the two groups. 

Both groups of parents reported similar levels of anxiety with respect to how rushed they felt during children’s mealtimes, and control parents often reported noisier mealtimes than parents of PKU children, although no differences were statistically significant between groups or over time.

### 3.2. Feed-Time Questionnaire

When asked how they felt before giving their child a solid meal during weaning, both groups scored low (between 1.4–2.0 out of 7), suggesting few negative feelings about solid food administration. Mothers of children with PKU, were well supported and at weaning were less anxious than controls (score 1.4 versus 2.0), but after an initial increase at eight months, their concerns had little change until a peak at two years of age. Control mothers became more confident about giving solid meals with increasing age of their children, although there was a peak again at two years of age. However, differences between PKU and control groups did not reach statistical significance, nor did differences within groups over time.

**Figure 2:**
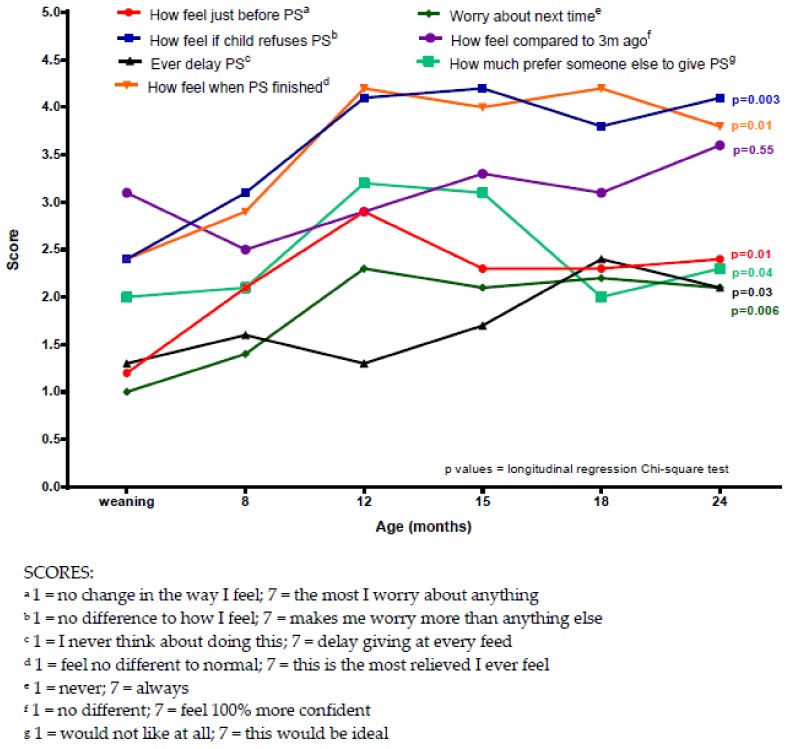
Mean maternal feelings about giving protein substitute to their child with phenylketonuria (PKU) at different child ages in the first two years.

When mothers of children with PKU were asked how they felt about various aspects of giving the protein substitute, concerns were generally low (scoring between 1 and 4.5 out of 7) but peaked at around 12 months of child age, then mostly plateauing between 12–24 months (Figure 2). When analysed using longitudinal regression, all questions showed a significant difference over time, with the exception of how they felt compared to three months ago. In particular, over time, child refusal of the protein substitute caused the most concern (*p* = 0.003), and mothers expressed relief post protein substitute administration (*p* = 0.01) (Figure 2).

**Figure 3:**
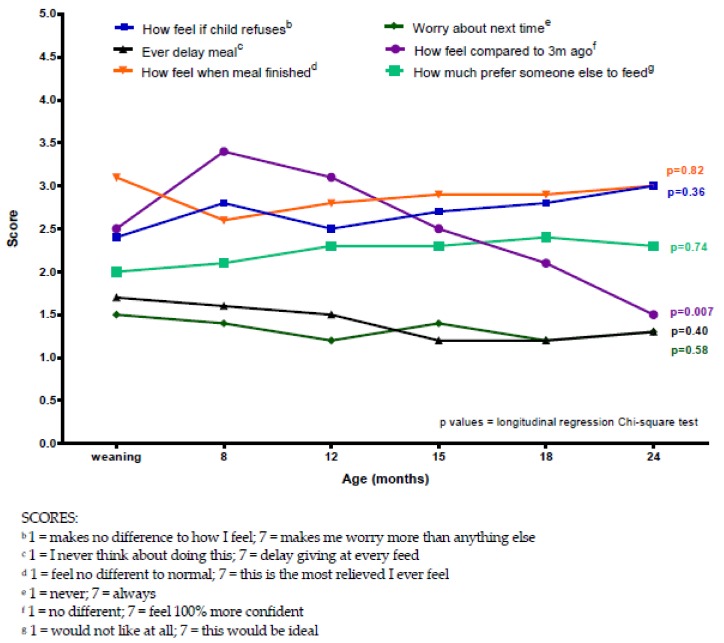
Mean control maternal feelings about feeding their child solid foods at different child ages in the first two years.

However, when control mothers were asked the same questions but with respect to feeding their child solid foods, there was less change over time, with mean scores for each question remaining comparable across all assessment ages (Figure 3) and lower (scores between 1 and 3.5) than in the PKU group. The exception being the question about how they felt compared to three months ago (*p* = 0.007), which had a sharp increase in anxiety score between weaning commencement and eight months, followed by a gradual decline, suggesting that they were coping better with weaning over time. 

### 3.3. Beck Anxiety Inventory (BAI)

**Figure 4:**
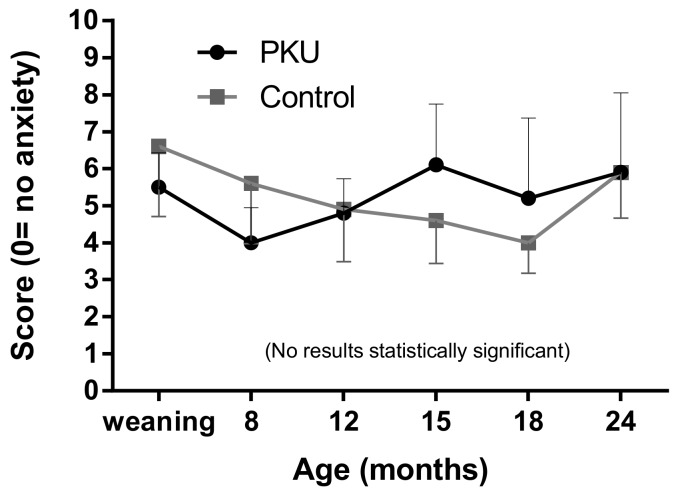
Mean anxiety scores for mothers of children with PKU and control mothers.

Mothers of both groups of children demonstrated low levels of anxiety with mean scores below 7 (minimal anxiety) at all ages (Figure 4). However, whilst there was a gradual decline in mean anxiety in the control group with a spike again at two years, the mothers of children with PKU showed more variable anxiety (larger standard deviations), peaking at 15 months and remaining higher than control mothers until two years, when both groups had equivalent levels of anxiety. Differences were not statistically significant between groups or over time. 

**Figure 5:**
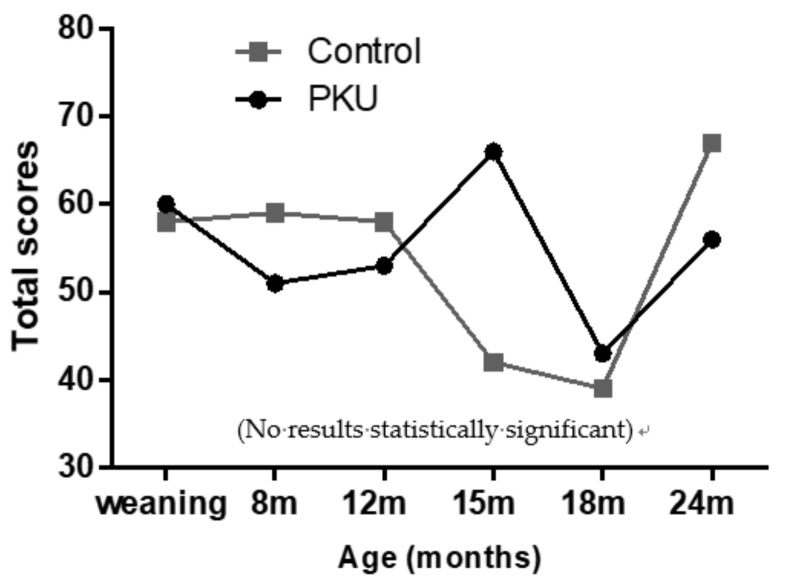
Total group scores for emotive symptoms experienced by mothers of PKU children and control mothers.

Due to the predominantly physiological focus of symptoms in the Beck’s questionnaire, emotional/cognitive symptoms (nervous, terrified/afraid, fear of losing control, fear of dying, fear of the worst happening and inability to relax) were separated for analysis. When grouped together, and individual scores totalled (1 = mildly, 2 = moderately, 3 = severely) for all emotive symptoms, there was no significant difference between the two study groups (Figure 5). However, mothers of children with PKU had a peak score at 15 months of age, and this was notably higher than observed in controls, although not statistically significant. Conversely, mothers of control children had peak scores at 24 months.

### 3.4. The Coping Health Inventory for Parents (CHIP) Questionnaire

When compared with normative mean data [11], results on the CHIP questionnaire showed that mothers of children with PKU scored, on average, higher in all three subsets (Table 1), meaning that they utilise coping behaviours more so than the average mother of a child with a chronic illness. Highest scores were at weaning, 15 and 24 m in all three subsets. Coping strategies were significantly higher at both 15 and 24 m, compared with 18 m in subset B (15 versus 18 m, *p* = 0.04 and 18 versus 24 m, *p* = 0.02; paired *t*-test) and at 15 m compared with 8 m in subset C (*p* = 0.05; paired *t*-test). Frequency of using the three different coping strategies/subsets varied, with mothers of children with PKU using family integration, cooperation and optimism (subset A), and medical communication and consultation (subset C) significantly more often than social support, self-esteem and psychological stability (subset B). Out of a total score of 3, individual mean scores for subset A were 2.3, for subset C were 2.2 and subset B was significantly lower at 1.8 (Table 1). 

Control mothers in comparison scored lower on subsets A and B than mothers of children with PKU (subset C was not compared as it was largely based on behaviours related to medical care; questions directly relating to medical conditions in subset A were also removed for comparative analysis) (Table 2). Differences were statistically significant at 15 and 24 months of age for subset A, and at weaning and 15 months for subset B. Longitudinal regression analysis demonstrated that there was a significant difference between groups for both subset A and subset B (*p* < 0.0001), but this difference did not change overtime between the PKU and control groups. 

When considering individual coping behaviours, a large percentage (95%) of mothers of children with PKU reported behaviours that related to medical care as being most helpful, particularly at weaning age. For example, the most helpful coping behaviour in subset A for the PKU group was believing that their child was getting the best medical care (95% scoring it a 3), followed by doing things with their children, talking to medical staff, ensuring medical treatments are carried out at home and trusting in their spouse for support. Whilst for the control group, doing things with their children was reported as the most helpful (65% scoring it a 3), followed by trusting their spouse for support, doing things together as a family and building a closer relationship with their spouse. In subset B, the most helpful behaviour in both groups was sleeping (70% of PKU and 50% of controls scoring it a 3). Other behaviours that scored highly were similar across both PKU and control groups, although the PKU group generally had a greater number of respondents scoring 3 for all behaviours. In contrast, the coping behaviours considered least helpful (score of 0) by the largest percentage of mothers (up to 50%) were similar across groups, generally from subset B, and focused on working and outside employment and doing things for themselves (hobbies, entertaining, getting away on their own). 

### 3.5. Blood Phenylalanine Levels

**Figure 6:**
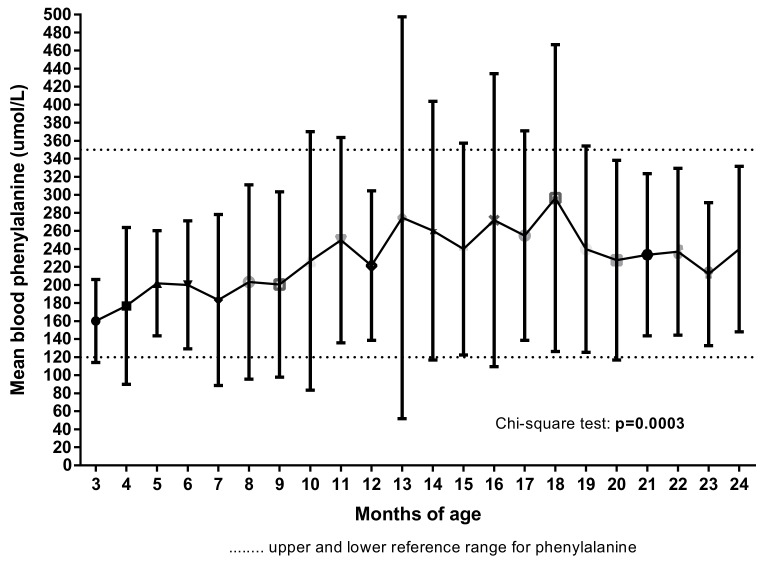
Mean child blood phenylalanine levels by age.

Mean blood phenylalanine levels were highest and more variable between 13 to 18 months of age (*p* = 0.04, analysis of variance (ANOVA)) (Figure 6) in line with levels of maternal stress, anxiety and coping behaviours. Longitudinal regression also demonstrated a significant change in Phe values over time (*p* = 0.0003).

In addition, there were correlations between higher blood Phe and lower scores for mealtime maternal stress (i.e., more stress) at 15 months (*p* = 0.01; r = −0.56; Spearman, 90% confidence interval (CI), 2-tailed) and 18 months (*p* = 0.03; r = −0.51) but not at other ages. Similarly, mothers’ mealtime mood was negatively correlated with blood Phe levels at 15 and 18 months (*p* = 0.04; r = −0.48) only. There was also a correlation between higher Phe levels and concern about child refusal of protein substitute at 15 months of age (*p* = 0.05; r = 0.44). 

## 4. Discussion

This is one of the first longitudinal feeding studies in PKU to report maternal anxiety and changes in coping strategies during the introduction of solid foods and second-stage protein substitute. It is important that this is documented. The same longitudinal study has already reported on the challenges of feeding children with PKU [13,14]. Before 12 months of age, weaning was associated with low levels of anxiety in both mothers of children with PKU and mothers of normal healthy children. When weaning first commenced, mothers of children with PKU were relaxed at mealtimes in contrast to control mothers who were more stressed at the start of weaning but became more confident after this initial feeding milestone had been reached. It was evident that support from family and specialist dietitians and confidence in their child’s medical care helped mothers cope with the demands of PKU management. However, as infants approach teething age and encounter minor illness and growing independence, feeding in PKU becomes more challenging and parents worry about the neurological consequences of poor dietary control. Overall, mothers of children with PKU were more variable with their concerns about feeding. This became particularly evident from 12 months of age, when their there were more issues, particularly with protein substitute administration. Results of four different study questionnaires consistently demonstrated that at around 15 months of child age, there were peaks in maternal stress and anxiety in mothers of children with PKU, and they had increased reliance on coping mechanisms. Conversely, control parents generally became more confident and less anxious with time but had peak concerns at two years of age, perhaps coinciding with growing child independence. 

In PKU, there is an association between parent stress, anxiety and depression [3]. Some studies report a higher incidence in parents (particularly mothers) of children with PKU compared with parents of healthy controls, negatively impacting on quality of life and psychosocial wellbeing [15], others report no significant difference [16,17]. Whilst this study found no overall differences in anxiety, there was a suggestion that mothers of children with PKU experienced more emotive or subjective symptoms (as opposed to physiological symptoms), specifically at 15 months of age, compared to controls. This is consistent with quality of life studies in PKU that report a greater emotional impact on parents [18]. Fear of neurological damage in their children dominates their thoughts [1,3], and it is established that average blood phenylalanine levels rise in the second year of life [19], associated with teething and intercurrent illnesses. In this study, blood phenylalanine levels were highest between 13 to 18 months of age, correlating with increased levels of maternal stress, particularly with respect to protein substitute refusal. This lends further support to the previously acknowledged age of feeding difficulty of 12–18 months in children with PKU [13]. It was reassuring in this study that by the age of two years, anxiety associated with feeding children with PKU was similar to control mothers. Throughout this time period, intense dietetic support and proactive management strategies to counteract feeding problems as they arise may have helped lessen parental anxiety.

Mothers of children with PKU frequently carry the responsibility for managing the low Phe diet, including meal preparation and dietary supervision. This requires an additional time burden (an average of 19 h/week extra) [15], which may result in a loss of earnings [1,6]. Fathers, in contrast, have been reported to feel less disease-specific parenting stress as they may be less directly involved in dietary management [15], and this disparity can affect relationships within the family [1,5]. Furthermore, low educational levels in parents have been reported to compound levels of stress and depression [2,15]. 

Mothers of children with PKU demonstrated significantly higher coping scores than control mothers at weaning, 15 and 24 months of age, higher scores compared to normative data for mothers of chronically ill children and similar scores to that previously reported for parents of children with asthma [20]. In theory, the more coping behaviours used by parents, the less stress they will experience, so higher CHIP scores reflect more adaptive styles of coping [11]. Therefore, in line with previous research into parental anxiety in PKU [17], mothers of children with PKU seem to adapt to the additional stresses that they experience during the weaning period. They are most likely to cope by relying on family (including grandparents) [4], being optimistic [3] and gaining understanding of PKU through communication with their specialist dietitians. This involved believing their children were receiving the best medical care, spending quality time with their children, receiving support from their spouse and trying to ensure prescribed protein substitute and protein exchanges were taken. They were less likely to use strategies that involved social support outside of the family or caring for their own self-esteem and psychological wellbeing by pursuing hobbies [1]. Whilst all mothers in this study had good support from a specialist dietitian, two thirds of mothers with PKU (65%) used the internet for support compared with 50% using their own family support network (e.g., grandparents) and 35% using other health professionals (health visitors, GPs, nurses). This is surprising but may reflect a need to talk to other mothers who have similar experiences. 

Therefore, following a diagnosis of PKU and early months of weaning, families need to redefine their way of life. Family unity, optimism in the face of stress, developing positive relationships with their health care team and gaining a good understanding of the treatment required are important coping mechanisms [3,4]. Regular weekly contact with a consistent and experienced dietitian for the first two years of life is central to this support. Maintaining weekly blood phenylalanine monitoring will provide knowledge and reassurance about blood Phe control. Encouraging guilt-free downtime for mothers to do things for themselves is important in improving self-confidence, enabling adaption to the changing family dynamics and ultimately, to cope better with their situation. However, it is acknowledged that this is difficult as mothers may fear relinquishing control and allowing others to care for their child, who they may not trust to administer the diet as prescribed [3,8]. Providing formal training about PKU, protein substitute preparation and food suitability to grandparents and other family members is important. Anxiety and stress may lead to parents using overprotective parenting strategies that restrict the child’s emotional or social development [3]. Assisting parents to maintain a balance of different coping behaviours is imperative.

There were some study limitations. While consistent trends were identified between PKU and control groups with respect to anxiety and coping, results in this study were often not statistically significant, probably associated with the small numbers of patients available for recruitment. A larger cohort of subjects may have led to more feasible statistical comparisons and more significant differences, and this is an area that warrants further study. Furthermore, the Beck’s anxiety inventory focused predominantly on physiological symptoms. In keeping with previous reports in parents of children with PKU, this study identified a more significant impact from emotional symptoms [3]. In addition, only mothers completed questionnaires, so coping strategies and anxiety may manifest in different ways in fathers. They may also differ according to the severity of PKU, the age of the child and country of origin. How tearful or happy children were during mealtimes was also a subjective measure that was likely influenced by the mother’s feelings at the time. It also must be noted that one parent in the control group had experienced weaning both a PKU and a non-PKU child (although they were only included in the control group and the PKU siblings were adults) and this may have influenced their responses. In addition, the feed-time questionnaire looked at feelings around protein substitute administration for parents of children with PKU, but for the control children, it looked at administration of solid food. It might have been useful to look at maternal feelings around solid food administration in the PKU group as well, to ascertain whether it is mostly administering the protein substitute that is causing anxiety or whether feeding solid food also causes additional stress. 

## 5. Conclusions

Mothers of children with PKU appear to cope well with weaning, with low levels of stress and anxiety comparable to control mothers. Administration of a protein substitute is challenging, particularly between 12–18 months of age. This is a key period during weaning when mothers of children with PKU feel most anxious and stressed, coinciding with raised blood phenylalanine levels, teething, illness and children developing their own independence. Health professionals need to be cognisant of this vulnerable period and be prepared to offer more directional support as required. Periodic psychological reviews of parental stress levels, anxiety and coping strategies would enable health professionals to better identify those parents of children with PKU most at risk of depression or negative psychosocial outcomes. However, it is reassuring to report that by two years of child age, with health professional support, this group of mothers of children with PKU reported similar levels of anxiety and stress to that of control mothers.

## Figures and Tables

**Table 1:** Means and standard deviations for phenylketonuria (PKU) coping health inventory for parents (CHIP) subsets compared with normative data.

	A. Family Integration, Cooperation, Optimism		B. Social Support, Self-Esteem, Psychological Stability		C. Medical, Communication, Consultation	
No. of Items	19		18		8	
	Mean Score (SD)	Mean Score per Item *	Mean (SD)	Mean Score per Item *	Mean (SD)	Mean Score per Item *
Normative data [11]	40 (15)		28 (12)		15 (7)	
PKU weaning	45 (9.0)	2.4	34 (8.6)	1.8	19 (4.6)	2.4
PKU 8 m	42 (7.6)	2.2	29 (11)	1.6	16 (5.7)	2.0
PKU 12 m	44 (8.0)	2.3	31 (11)	1.7	17 (4.5)	2.1
PKU 15 m	44 (6.5)	2.3	33 (8.6)	1.9	18 (4.5)	2.3
PKU 18 m	42 (9.6)	2.2	30 (8.6)	1.7	17 (4.8)	2.1
PKU 24 m	44 (7.3)	2.3	33 (9.7)	1.8	18 (4.5)	2.3
Mean	43 (8.0)	2.3	32 (9.6)	1.8	18 (4.8)	2.2

* Unpaired t-tests: A versus B *p* < 0.0001 for all ages; A versus C *p* = NS (not significant) for all ages; B versus C *p* = 0.0002 (weaning), *p* = 0.003 (8 m), *p* = 0.004 (12 m), *p* = 0.001 (15 m), *p* < 0.0001 (18 m), *p* = 0.003 (24 m). SD = standard deviation.

**Table 2:** Means and standard deviation scores for PKU CHIP subsets compared with control mothers.

Infant Age at Questionnaire Completion	A. Family Integration, Cooperation, Optimism			B. Social Support, Self-Esteem, Psychological Stability		
No. of Items	13			18		
	Mean (SD)			Mean (SD)		
	PKU **	Control	*p* Value *	PKU	Control	*p* Value *
**Weaning**	30 (6.4)	27 (6.9)	0.17	34 (8.6)	24 (10)	0.006
**8 m**	29 (5.5)	26 (7.0)	0.20	29 (11)	26 (11)	0.38
**12 m**	30 (6.4)	27 (6.5)	0.10	31 (11)	29 (11)	0.61
**15 m**	30 (4.9)	25 (7.9)	0.03	33 (8.6)	26 (11)	0.03
**18 m**	28 (7.0)	25 (7.3)	0.19	30 (8.6)	29 (10)	0.89
**24 m**	30 (5.8)	25 (6.7)	0.02	33 (9.7)	27 (9.4)	0.07

* unpaired *t*-test. ** Phenylketonuria.

## References

[1] Ford S., Driscoll M.O., MacDonald A. (2018). Living with Phenylketonuria: Lessons from the PKU community. Mol. Genet. Metab. Rep..

[2] Irannejad F., Dehghan M., Rabori M. (2018). Stress and quality of life in parents of children with phenylketonuria. J. Child Adolesc. Psychiatr. Nurs..

[3] Carpenter K., Wittkowski A., Hare D.J., Medford E., Rust S., Jones S.A., Smith D.M. (2018). Parenting a Child with Phenylketonuria (PKU): An Interpretative Phenomenological Analysis (IPA) of the Experience of Parents. J. Genet. Couns..

[4] Medford E., Hare D.J., Carpenter K., Rust S., Jones S., Wittkowski A. (2017). Treatment Adherence and Psychological Wellbeing in Maternal Carers of Children with Phenylketonuria (PKU). JIMD Rep..

[5] Awiszus D., Unger I. (1990). Coping with PKU: Results of narrative interviews with parents. Eur. J. Pediatr..

[6] MacDonald A., Smith T.A., de Silva S., Alam V., van Loon J.M. (2016). The personal burden for caregivers of children with phenylketonuria: A cross-sectional study investigating time burden and costs in the UK. Mol. Genet. Metab. Rep..

[7] Almeida A.C., Pereira M.G. (2016). Psychometric Properties of the Portuguese Version of the Coping Health Inventory for Parents (CHIP) of Adolescents With Chronic Illness. J. Pediatr. Nurs..

[8] Medical Research Council Working Party on Phenylketonuria (1993). Recommendations on the dietary management of phenylketonuria. Report of Medical Research Council Working Party on Phenylketonuria. Arch. Dis. Child..

[9] Grant M.M. (2011). Beck Anxiety Inventory. Encyclopedia of Child Behavior and Development.

[10] Beck A.T., Steer R.A. (1993). BAI: Beck Anxiety Inventory: Manual.

[11] Sclare I. (1997). The Child Psychology Portfolio.

[12] Evans S., Daly A., Wildgoose J., Cochrane B., Chahal S., Ashmore C., Loveridge N., MacDonald A. (2019). Growth, Protein and Energy Intake in Children with PKU Taking a Weaning Protein Substitute in the First Two Years of Life: A Case-Control Study. Nutrients.

[13] Evans S., Daly A., Wildgoose J., Cochrane B., Chahal S., Ashmore C., Loveridge N., MacDonald A. (2019). How Does Feeding Development and Progression onto Solid Foods in PKU Compare with Non-PKU Children During Weaning?. Nutrients.

[14] Evans S., Daly A., MacDonald J., Pinto A., MacDonald A. (2017). Fifteen years of using a second stage protein substitute for weaning in phenylketonuria: A retrospective study. J. Hum. Nutr. Diet..

[15] Gunduz M., Arslan N., Unal O., Cakar S., Kuyum P., Bulbul S.F. (2015). Depression and anxiety among parents of phenylketonuria children. Neurosciences.

[16] Kazak A.E., Reber M., Snitzer L. (1988). Childhood chronic disease and family funcitoning: A study of phenylketonuria. Pediatrics.

[17] Ambler O., Medford E., Hare D.J. (2018). Parenting a Child with Phenylketonuria: An Investigation into the Factors That Contribute to Parental Distress. JIMD Rep..

[18] Bosch A.M., Burlina A., Cunningham A., Bettiol E., Moreau-Stucker F., Koledova E., Benmedjahed K., Regnault A. (2015). Assessment of the impact of phenylketonuria and its treatment on quality of life of patients and parents from seven European countries. Orphanet J. Rare Dis..

[19] MacDonald A., Davies P., Daly A., Hopkins V., Hall S.K., Asplin D., Hendriksz C., Chakrapani A. (2008). Does maternal knowledge and parent education affect blood phenylalanine control in phenylketonuria?. J. Hum. Nutr. Diet..

[20] Garro A. (2011). Coping patterns in Latino families of children with asthma. J. Pediatr. Health Care.

